# Predicting in-hospital cardiac arrest outcomes: CASPRI and GO-FAR scores

**DOI:** 10.1038/s41598-023-44312-2

**Published:** 2023-10-23

**Authors:** Jonghee Jung, Ji Ho Ryu, Seungwoo Shon, Munki Min, Tae Gyu Hyun, Mose Chun, Daesup Lee, Minjee Lee

**Affiliations:** 1 Department of Emergency Medicine, Pusan National University Yangsan Hospital, Kumoh-ro 20, Mulgum-up, Yangsan-si, Gyeongsangnam-do 50612 Korea; 2 Present Address: Department of Emergency Medicine, Pusan National University School of Medicine, Yangsan-si, Gyeongsangnam-do 50612 Korea

**Keywords:** Medical research, Neurology, Risk factors, Signs and symptoms

## Abstract

It is important to predict the neurological prognoses of in-hospital cardiac arrest (IHCA) patients immediately after recovery of spontaneous circulation (ROSC) to make further critical management. The aim of this study was to confirm the usefulness of the Cardiac Arrest Survival Post-Resuscitation In-hospital (CASPRI) and Good Outcome Following Attempted Resuscitation (GO-FAR) scores for predicting the IHCA immediately after the ROSC. This is a retrospective analysis of patient data from a tertiary general hospital located in South Korea. A total of 488 adult patients who had IHCA and achieved sustained ROSC from September 2016 to August 2021 were analyzed to compare effectiveness of the CASPRI and GO-FAR scores related to neurologic prognosis. The primary outcome was Cerebral Performance Category (CPC) score at discharge, defined as a CPC score of 1 or 2. The secondary outcomes were survival-to-discharge and normal neurological status or minimal neurological damage at discharge. Of the 488 included patients, 85 (20.8%) were discharged with good prognoses (CPC score of 1 or 2). The area under the receiver operating characteristic curve of CASPRI score for the prediction of a good neurological outcome was 0.75 (95% CI 0.69–0.81), whereas that of GO-FAR score was 0.67 (95% CI 0.60–0.73). The results of this study show that these scoring systems can be used for timely and satisfactory prediction of the neurological prognoses of IHCA patients after ROSC.

## Introduction

In-hospital cardiac arrest (IHCA) is a major public health problem. IHCA occur 9–10 cases per 1000 admissions^1^. It is much more common than what we expected. IHCA is directly associated with morbidity and mortality. Compared with out-of-hospital cardiac arrest (OHCA), most cases of IHCA can be predicted and prevented because inpatients with comorbidities and in deteriorating clinical situations are constantly monitored^2^. Nevertheless, interest in IHCA is low compared to OHCA or high-risk cardiovascular disorders, such as myocardial infarction and stroke. Owing to early detection and advanced resuscitation, return of spontaneous circulation (ROSC) is achieved in 70–75% of IHCA cases^3^. However, the survival-to-discharge rate of IHCA ranges from 20 to 25%, which is considerably low^4^. This is because as patients with various medical complications are usually hospitalized, IHCA generally occurs in patients in a clinically deteriorated state. Therefore, failure of various organs, accompanied by neurological conditions and complications, must be considered in cases of IHCA^5^.

The Cardiac Arrest Survival Post-Resuscitation In-hospital (CASPRI) score is a validation tool proposed by Chan et al. in 2012. The CASPRI score includes important factors in the survival chain of cardiopulmonary resuscitation (CPR), such as the patient’s initial rhythm, time to defibrillation, and CPR execution time, and reflects their impact on survival and prognosis. In addition, the scoring system reflects the situation at the time CPR was performed and the patient’s clinical condition^6^, (Table S1). The Good Outcome Following Attempted Resuscitation (GO-FAR) score was developed using the large Get with the guidelines-Resuscitation database in the United States, which included 51,240 patients from 366 hospitals who had IHCA between 2007 and 2009^7,8^ (Table S2, S3). The GO-FAR score was created by emphasizing various underlying diseases and clinical problems in comparison with the patient's CASPRI score. The CASPRI and GO-FAR scores are indicators that reflect a patient's comorbid clinical diseases and cardiac arrest status and are useful tools for predicting a patient's prognosis shortly after cardiac arrest. The aim of this study was to confirm the usefulness of the CASPRI and GO-FAR scores as tools for predicting the neurological prognoses of IHCA patients immediately after ROSC.

## Results

### Baseline characteristics and clinical outcomes

**Figure 1:**
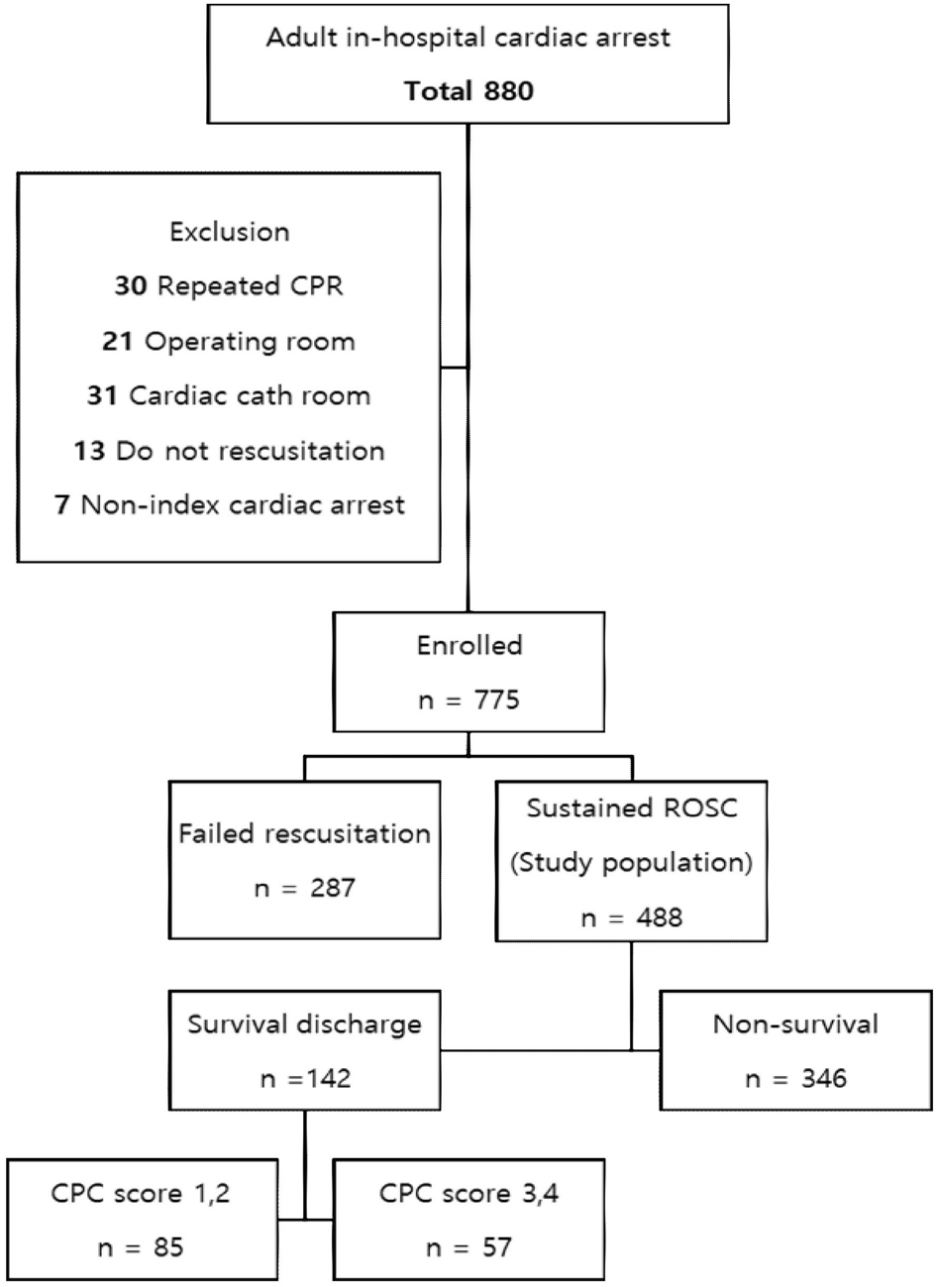
A flowchart of baseline characteristics and outcomes. *CPR* cardiopulmonary resuscitation, *ROSC* return of spontaneous circulation, *CPC* cerebral performance category.

Among 1107 patients with in-hospital cardiac arrest, 332 were excluded based on criteria. Of the remaining 775 patients aged 18 or older, 488 (62.97%) achieved sustained ROSC for 20 min. Out of the 488 patients, 142 survived until hospital discharge, resulting in an overall survival-to-discharge rate of 29.18%. Eighty-five patients (10.97% of the adult IHCA patients) were discharged with a Cerebral Performance Categories score (CPC score) of 1 or 2 (Fig. 1). The overall median age of the patients was 67 years, whereas that of patients with a CPC score of 1 or 2 and a CPC score of 3, 4, or 5 was 65 years and 67 years, respectively. 55.94% of the patients were hospitalized for non-cardiac medical illnesses. Non-cardiac causes accounted for approximately 76% of all cases of cardiac arrest. In addition, the neurological prognoses of patients hospitalized for non-cardiac causes were poorer than those of patients hospitalized for surgical or cardiac causes (p < 0.001). Overall, CPR was performed for an average of 12.91 min. For patients with a CPC score of 1 or 2, CPR was performed for an average of 7.88 min. For patients with poor neurological scores, CPR was performed for an average of 14.00 min (*p* < 0.001). A patient's neurological score before cardiac arrest, including CPC score at admission and 24 h prior to cardiac arrest, influenced the neurological status at hospital discharge (*p* < 0.001). More than 50% of patients in the poor outcome groups underwent early withdrawal of life-sustaining treatment (early WLST) or Do-Not-Resuscitation (DNR) order within 72 h after ROSC (*p* < 0.001). The time of cardiac arrest, place of occurrence, initial rhythm, underlying disease of the patient, and the accompanying clinical situation at the time of cardiac arrest are summarized in Tables 1 and 2.

**Table 1:** Baseline characteristics of patients according to neurological outcomes at hospital discharge.

	Total (n = 488)	Good (n = 85)^a^	Poor (n = 403)^b^	*p*
Age (median, IQR)	67.0 [58.00;76.00]	65.0 [56.00;75.00]	67.0 [58.00;77.00]	0.098*
Sex, male	296 (60.7%)	50 (58.8%)	246 (61.0%)	0.704
Weekend, or holiday	145 (29.7%)	21 (24.7%)	124 (30.8%)	0.298
Time, night	158 (32.4%)	22 (25.9%)	136 (33.7%)	0.202
Location				0.574
Ward	163 (33.4%)	26 (30.6%)	137 (34.0%)	
Telemetry unit	126 (25.8%)	20 (23.5%)	106 (26.3%)	
ICU	199 (40.8%)	39 (45.9%)	160 (39.7%)	
Illness category				< 0.001
Noncardiac, medical	273 (55.9%)	30 (35.3%)	243 (60.3%)	
Cardiac, medical	116 (23.8%)	20 (23.5%)	96 (23.8%)	
Noncardiac, surgical	71 (14.6%)	20 (23.5%)	51 (12.7%)	
Cardiac, surgical	28 (5.7%)	15 (17.6%)	13 (3.2%)	
Diabetes mellitus	189 (38.7%)	39 (45.9%)	150 (37.2%)	0.048†
MI, this admission	62 (12.7%)	20 (23.5%)	42 (10.4%)	0.002†
Respiratory insufficiency	297 (60.9%)	44 (51.8%)	253 (62.8%)	0.067†
Pneumonia	248 (50.8%)	29 (34.1%)	219 (54.3%)	0.001†
Renal insufficiency	192 (39.3%)	33 (38.8%)	159 (39.5%)	1.000†
Malignancy	142 (29.1%)	9 (10.6%)	133 (33.0%)	< 0.001†
Hepatic insufficiency	161 (33.0%)	27 (31.8%)	134 (33.3%)	0.899†
Sepsis	161 (33.0%)	20 (23.5)	141 (35.0%)	0.043†
Major trauma	17 (3.5%)	1 (1.2%)	16 (4.0%)	0.329†
Acute stroke	40 (8.2%)	4 (4.7%)	36 (8.9%)	0.276†
Transfer from nursing facility	61 (12.5%)	4 (4.7%)	57 (14.2%)	0.018†
Mechanical ventilation	160 (32.8%)	29 (34.1%)	131 (32.5%)	0.800†
Use of vasopressors	192 (39.3%)	31 (36.5%)	161 (40.0%)	0.625†
Acute CNS depression	178 (36.5%)	23 (27.1%)	155 (38.5%)	0.048†
CPC score at admission				0.048
CPC 1	213 (43.7%)	45 (52.9%)	168 (41.7%)	
CPC 2	151 (30.9%)	24 (28.2%)	127 (31.5%)	
CPC 3	80 (16.4%)	16 (18.8%)	64 (15.9%)	
CPC 4	44 (9.0%)	0 (0.0%)	44 (10.9%)	
CPC score before 24 h				< 0.001
CPC 1	92 (18.9%)	31 (36.5%)	61 (15.1%)	
CPC 2	184 (37.7%)	30 (35.3%)	154 (38.2%)	
CPC 3	162 (33.2%)	23 (27.1%)	139 (34.5%)	
CPC 4	50 (10.2%)	1 (1.2%)	49 (12.2%)	

*ICU* intensive care unit, *MI* myocardial infarction, Malignancy: active metastatic or hematologic cancer, *CPC* cerebral performance category score.

*Data are expressed as median, (interquartile range, IQR).

^†^Fisher’s exact test.

^a^Good neurological outcome: defined as CPC 1 or 2.

^b^Poor neurological outcome: defined as CPC 3 to 4, or death.

**Table 2:** Factors associated with CPR and neurological outcome: defined as CPC score.

	Total (n = 488)	Good (n = 85)^a^	Poor (n = 403)^b^	*P*
Arrest cause				< 0.001
Noncardiac	371 (76.0%)	45 (52.9%)	326 (80.9%)	
Cardiac	108 (22.1%)	39 (45.9%)	69 (17.1%)	
Unknown	9 (1.8%)	1 (1.2%)	8 (2.0%)	
Initial rhythm				< 0.001
VF/pulseless VT	94 (19.3%)	28 (32.9%)	66 (16.4%)	
PEA	269 (55.1%)	45 (52.9%)	223 (55.6%)	
Asystole	125 (25.6%)	12 (14.1%)	113 (28.0%)	
CPR duration (median, IQR)	10.0 [5.0;16.0]	5.0 [2.5;9.0]	10.0 [5.0;17.0]	< 0.001
Early WLST	209 (42.8%)	3 (3.5%)	206 (51.1%)	< 0.001†
CASPRI score				< 0.001
0 to 9	36 (7.4%)	13 (15.3%)	23 (5.7%)	
10 to14	204 (41.8%)	48 (56.5%)	156 (38.7%)	
15 to 19	146 (29.9)	16 (18.8)	130 (32.3)	
≥ 20	102 (20.9)	8 (9.4)	94 (23.3)	
CASPRI (median, IQR)	23.0 [19.0–28.0]	17.0 [13.5–22.5]	24.0 [20.0–28.0]	< 0.001
GO-FAR score				< 0.001
− 15 to − 6	36 (7.4%)	13 (15.3%)	23 (5.7%)	
− 5 to 13	204 (41.8%)	48 (56.5%)	156 (38.7%)	
14 to 23	146 (29.9%)	16 (18.8%)	130 (32.3%)	
≥ 24	102 (20.9%)	8 (9.4%)	94 (23.3%)	
GO-FAR (median, IQR)	14.0 [4.0–22.0]	6.0 [− 2.5–15.0]	15.0 [6.0–23.0]	< 0.001
MEWS_8hr (median, IQR)	3.0 [1.0;4.0]	2.0 [1.0;3.0]	3.0 [2.0;5.0]	< 0.001
qSOFA_8hr (median, IQR)	1.0 [0.0;2.0]	1.0 [0.0;1.0]	1.0 [0.0;2.0]	0.002

*VF* ventricular fibrillation, *VT* ventricular tachycardia, *PEA* pulseless electrical activity, *CPR* cardiopulmonary resuscitation. Early *WLST* withdrawal life sustaining treatment within 72 h after ROSC, *CASPRI* cardiac arrest survival post-resuscitation in-hospital, *GO-FAR* good outcome-following attempted resuscitation, *MEWS* modifeied early warning score, *qSOFA* quick sepsis related organ failure.

*Data are expressed as median, (interquartile range, IQR).

^†^Fisher’s exact test.

^a^Good neurological outcome: defined as CPC 1 or 2.

^b^Poor neurological outcome: defined as CPC 3 to 4, or death.

### The efficacy of the CASPRI and GO-FAR scores for the prediction of neurological prognosis.

**Figure 2:**
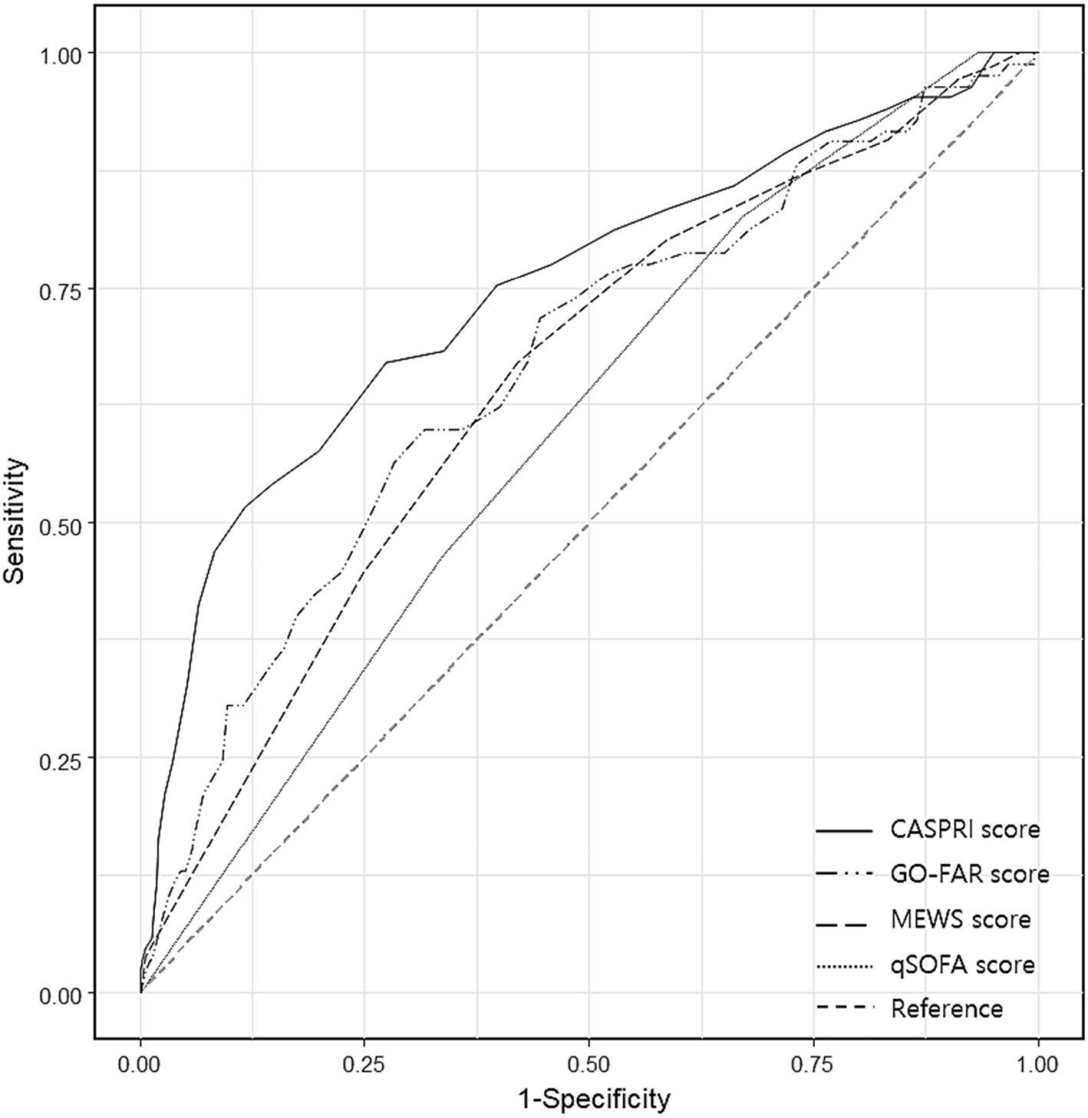
ROC curve to predict poor neurological outcome in IHCA. CASPRI score: AUC 0.75 (95% CI 0.69–0.81), GO-FAR score: AUC 0.67 (95% CI 0.60–0.73), MEWS score 8 h before cardiac arrest: AUC 0.65 (95% CI 0.59–0.72), qSOFA score 8 h before cardiac arrest: AUC 0.60 (95% CI 0.54–0.66). ROC: receiver operating characteristics, IHCA: In-hospital cardiac arrest, AUC: area under curve, CPC score: cerebral performance category, CASPRI: cardiac arrest survival post-resuscitation in hospital, GO-FAR: good outcome following attempted resuscitation, MEWS: modified early warning score, qSOFA: quick sequential organ failure assessment.

**Figure 3:**
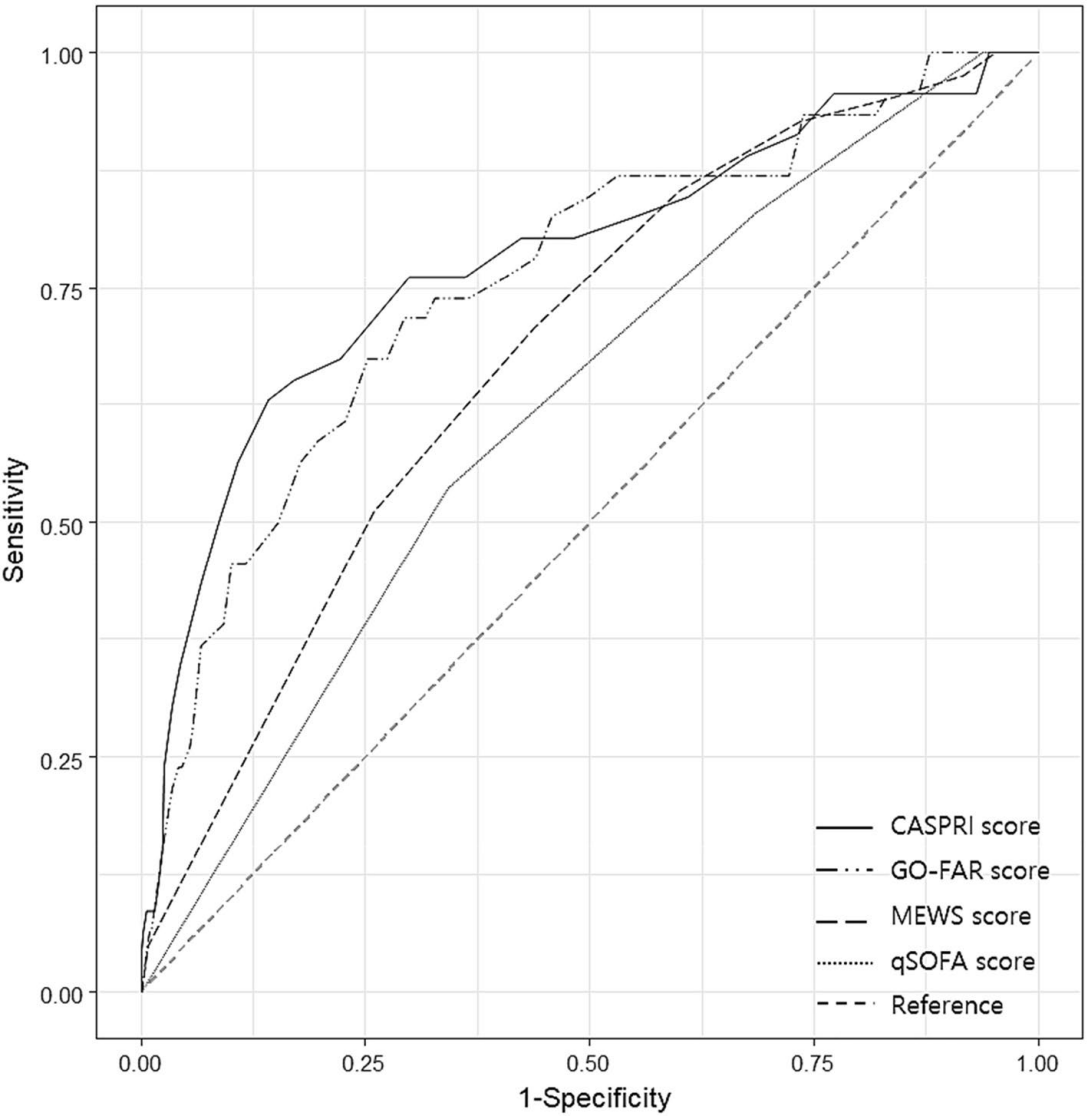
ROC curve for neurologic outcome other than neurologically intact discharge (CPC1) in IHCA. CASPRI score: AUC 0.78 (95% CI 0.70–0.86) GO-FAR score: AUC 0.76 (95% CI 0.68–0.84), MEWS score 8 h before cardiac arrest: AUC 0.68 (95% CI 0.60–0.76), qSOFA score 8 h before cardiac arrest: AUC 0.62 (95% CI 0.54–0.70). ROC: receiver operating characteristics, IHCA: In-hospital cardiac arrest, AUC: area under curve, CPC score: cerebral performance category, CASPRI: cardiac arrest survival post-resuscitation in hospital, GO-FAR: good outcome following attempted resuscitation, MEWS: modified early warning score, qSOFA: quick sequential organ failure assessment.

**Figure 4:**
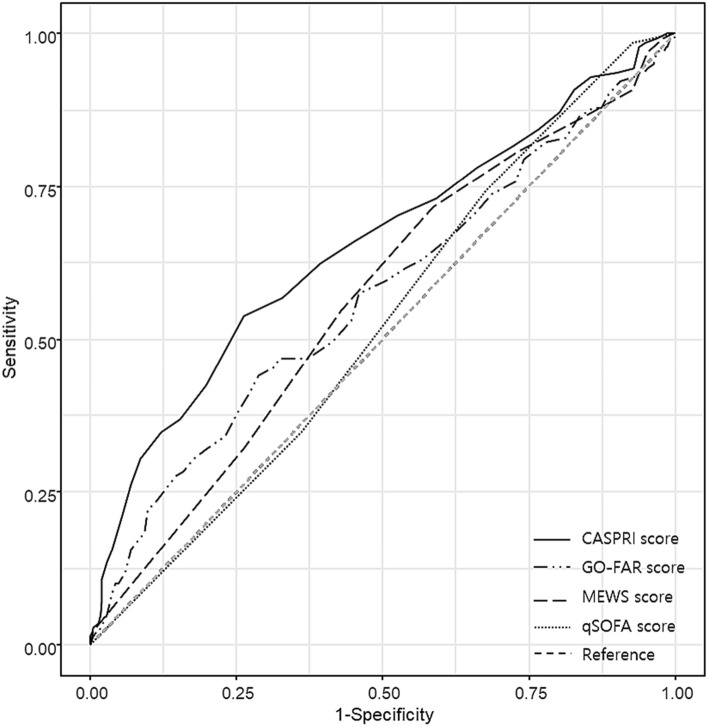
ROC curve for survival discharge in IHCA. CASPRI score: AUC 0.65 (0.60–0.71), Go-FAR score: AUC 0.57 (0.51–0.63), MEWS score before. 8 h: AUC 0.57 (0.51–0.62). qSOFA score before 8 h: AUC 0.52 (0.47–0.58). ROC: receiver operating characteristic, IHCA: In-hospital cardiac arrest, AUC: area under curve. CASPRI: cardiac arrest survival post-resuscitation in-hospital, GO-FAR: good outcome-following attempted resuscitation, MEWS: modifeied early warning score, qSOFA: quick sequential organ failure assessment.

The CASPRI score had an overall median of 23.0 points. Patients with CPC scores of 1 or 2 had a median CASPRI score of 17.0 points, while patients with CPC scores of 3, 4, or 5 had 24.0 points. The overall median GO-FAR score was 14.0. Patients with CPC scores of 1 or 2 had a lower median GO-FAR score of 6.0 points, while patients with CPC scores of 3, 4, or 5 had a higher mean GO-FAR score of 15.0 points. The results of the independent t-test showed that the CASPRI and the GO-FAR scores of patients with and without a good neurological prognosis were significantly different (both *p* < 0.001), (Table 2). The Area Under Receiver Operating Characteristic curve (AUROC) of CASPRI score was 0.75 (95% Confidence interval, 95% CI 0.69–0.81) and that of GO-FAR score was 0.67 (95% CI 0.60–0.73). The cutoff value for CASPRI score was 17 and that of GO-FAR was 9. Comparison of the predictive powers of the scores using Delong's test showed that CASPRI score had significantly higher predictive power than GO-FAR score (*p* = 0.018), Modified Early Warning Score (MEWS) (*p* = 0.019), and Sequential Organ Failure Assessment (qSOFA) (*p* < 0.001) (Fig. 2). For the prediction of discharge without neurological damage, the AUROC of CASPRI score was 0.78 (95% CI 0.70–0.86), whereas that of GO-FAR score was 0.76 (95% CI 0.68–0.84). However, although there were no significant differences between them (*p* = 0.557) (Fig. 3). Contrary to neurological prognosis at discharge, the CASPRI and GO-FAR scores exhibited limited predictive capability for survival discharge outcomes (Fig. 4). Although not shown in the table, the early WLST group had a median CASPRI score of 25, while the group without early WLST had a score of 22 (*p* =  < 0.001). Among those with CASPRI scores of 0–9 (indicating a favorable neurological prognosis), only 15.4% received early WLST, with 53.8% of them being discharged with CPC scores of 1 or 2. In contrast, for those with scores of 25 or higher, 51.2% underwent early WLST, but only 7.7% showed a favorable neurological prognosis. However, no significant difference in GO-FAR scores existed between the two WLST groups (*p* = 0.164).

### Factors related to the good neurological outcomes in IHCA

The multivariate logistic regression analysis of factors related to the neurological outcomes of IHCA showed that older age, longer CPR time, and comorbid malignancy or pneumonia resulted in significantly poor neurological prognosis. In addition, the results showed that the CPC score 24 h before cardiac arrest was related to the prognosis. Cardiac causes of cardiac arrest had a higher odds ratio (OR 4.09, 95% CI 2.48–6.76) in univariate analysis. However, in multivariable analysis, which considered various complex factors such as arrhythmias, underlying patient conditions, and the presence of defibrillation, the odds ratio was 1.52 (95% CI 0.56–4.11). Patients who underwent early WLST exhibited poor neurological outcomes (OR, 0.04; 95% CI 0.01–0.15). (Table 3).

**Table 3:** Univariable and multivariable logistic regression analysis for predictors of favorable neurologic outcome.

	Univariable		Multivariable	
	Odds ratio (95% CI)	*p*	Odds ratio (95% CI)	*p*
Age (year)	0.98 (0.97–1.00)	0.064	0.97 (0.95–1.00)	0.036
Illness category				
Non cardiac, medical	Reference			
Cardiac, medical	1.69 (0.91–3.12)	0.094	1.90 (0.87–4.12)	0.107
Non cardiac, surgical	3.18 (1.67–6.03)	< 0.001	1.10 (0.43–2.80)	0.841
Cardiac, surgical	9.35 (4.06–21.52)	< 0.001	1.29 (0.42–3.95)	0.656
CPR duration (minutes)	0.93 (0.90–0.97)	< 0.001	0.92 (0.88–0.96)	< 0.001
Initial rhythm				
Asystole	Reference			
PEA	1.89 (0.96–3.72)	0.064	1.62 (0.73–3.61)	0.237
VF/pulseless VT	3.40 (1.90–8.38)	< .001	3.16 (1.20–8.30)	0.020
Cause of arrest, cardiac	4.09 (2.48–6.76)	< .001	1.52 (0.56–4.11)	0.408
CPC score before 24 h				
CPC1	Reference			
CPC2	0.38 (0.21–0.69)	< 0.001	0.45 (0.20–1.00)	0.051
CPC3	0.33 (0.18–0.60)	< 0.001	0.32 (0.14–0.76)	0.010
CPC4	0.04 (0.01–0.30)	0.002	0.02 (0.00–0.18)	< 0.001
DM	1.61 (1.00–2.57)	0.049	1.39 (0.75–2.58)	0.295
MI, this admission	2.41 (1.32–4.39)	0.004	1.01 (0.33–3.06)	0.988
HF, this admission	2.89 (1.78–4.72)	< 0.001	1.26 (0.52–3.04)	0.610
Arrhythmia	2.73 (1.65–4.51)	< 0.001	2.25 (1.10–4.61)	0.027
Malignancy	0.21 (0.10–0.45)	< 0.001	0.33 (0.14–0.78)	0.012
Pneumonia	0.46 (0.29–0.75)	0.002	0.47 (0.25–0.89)	0.019
Acute stroke	0.36 (0.11–1.20)	0.097	0.51 (0.12–2.09)	0.348
Sepsis	0.57 (0.33–0.98)	0.043	0.88 (0.43–1.78)	0.712
Chronic respiratory disease	0.57 (0.31–1.05)	0.071	0.66 (0.29–1.50)	0.319
Respiratory failure	0.64 (0.40–1.02)	0.060	1.46 (0.73–2.95)	0.288
Acute CNS depression	0.59 (0.35–0.99)	0.049	0.54 (0.28–1.04)	0.068
Admission from nursing facility	0.030 (0.11–0.85)	0.023	0.75 (0.21–2.64)	0.650
Early WLST	0.04 (0.01–0.11)	< 0.001	0.04 (0.01–0.15)	< 0.001

Hosmer and Lemeshow goodness-of-fit Chi-Squared test* p* = 0.421, Nagelkerke’s R^2^ = 0.487.

*CI* confidence interval, *CPR* cardiopulmonary resuscitation, *VF* ventricular fibrillation, *VT* ventricular tachycardia, *PEA* pulseless electrical activity, *CPC* cerebral performance category score, *DM* diabetes melitus, *MI* myocardial infarction, *HF* heart failure, Chronic respiratory disease(such as chronic obstructive lung disease, asthma, interstitial lung disease, etc.), Acute CNS depression: 4 h before cardiac arrest, not stroke, Early WLST: Withdrawal Life Sustaining Treatment within 72 h after ROSC.

## Discussion

The neurological outcome following IHCA is a crucial concern for patients' guardians as it guides subsequent treatment decisions and influences a patient's quality of life. We found notable distinctions in CASPRI and GO-FAR scores between groups with favorable and unfavorable neurological prognoses. Specifically, CASPRI demonstrated a moderate predictive ability (AUROC: 0.75, 95% CI 0.69–0.81), indicating a reasonably effective model for neurological prognosis, whereas GO-FAR exhibited a lower predictive power (AUROC: 0.67, 95% CI 0.60–0.73) for assessing neurological prognosis. Although the comparison of AUCs by DeLong's test yielded a *p* value less than 0.05, indicating a significant difference between CASPRI and GO-FAR. Yet, their overlapping confidence intervals make it challenging to conclude that CASPRI significantly outperforms GO-FAR. Chan et al.^6^ who proposed the CASPRI score in 2012, suggested that a score of 10 or less could predict a good neurological prognosis. The model c-statistic of the multi-variate predictors of the scoring model was 0.80. In a study conducted by Wang et al. at a university hospital in Taiwan, the AUROC of CASPRI score was 0.79 and the median CASPRI score that could predict a CPC score of 1 or 2 was 17 (IQR 12.3–22)^9^. Chou et al. and Che-Hung et al. have conducted studies to investigate the usefulness of the CASPRI score for IHCA in the emergency department. The AUROC was 0.81 in the study conducted by Chou et al.^10^ and 0.77 in the study conducted by Tsai et al.^11^ In the present study, the AUROC of CASPRI score was 0.76, while slightly lower than some prior studies. However, the AUROC of CASPRI score for the prediction of a CPC score of 1 (discharge without neurological damage) was 0.78, indicating better predictive power for this outcome. Most of the studies are focused on GO-FAR score for the prediction of a CPC score of 1 at discharge. In the study by Thai et al. and those of the original United States cohort, a Stockholm country cohort, and a Swedish cohort, the AUROCs of GO-FAR score ranged from 0.75 to 0.85^12–14^. In the present study, the AUROC of GO-FAR score for the prediction of a CPC score of 1 at discharge was 0.76 (95% CI 0.68–0.84). Notably, Cho et al.'s study had distinct results and objectives. The study by Cho et al. is the only study in which a CPC score of 1 or 2 discharge was used as a primary outcome. That study was a single-center retrospective study conducted in Korea, under conditions comparable to this research. The survival-to-discharge rate in the study by Cho et al. was 25.4% and the percentage of the study population that was discharged with a good neurological prognosis (CPC score of 1 and 2) was 16.0%, which are different from the results of the present study., with a higher AUROC for GO-FAR in predicting poor neurological outcomes at discharge (0.81)^15^.

Moreover, our analysis highlighted several factors associated with positive neurological outcomes, such as younger age, cases originating from cardiac causes, and presence of arrhythmias. Conversely, the decision for early withdrawal of life-sustaining treatment was correlated with poorer neurological outcomes. These insights provide valuable implications for prognostic assessment and decision-making in in-hospital cardiac arrest cases. In this study, higher CASPRI scores were indicative of poor prognosis, leading to a higher frequency of early WLST decisions. The study results suggest that CASPRI and GO-FAR scores are more effective in predicting neurological prognosis rather than survival outcomes. However, they are not very reliable predictors of survival-to-discharge. Therefore, these scores can be used as supplementary tools during discussions with patients about their prognoses. In the previous American Heart Association guidelines, neuro-prognostication was recommended after 72 h of spontaneous circulation recovery. However, in the 2020 revision, the recommendation changed to prognostic judgment after 72 h of reaching normothermia, following therapeutic hypothermia completion. This delayed judgment by 2–3 days^16^. Clinicians should avoid premature prognosis due to the potential impact of sedatives and muscle relaxants on prognostication^17^. Neurological examinations, such as electroencephalogram (EEG), Magnetic Resonance Imaging (MRI), Somatosensory Evoked Potentials (SSEPs), and neuron-specific enolase, along with various critical care indicators, are used to determine neurological prognosis after IHCA^16^. However, in many cases, these tests cannot be performed due to the patient's clinical condition, time constraints, hospital administration issues, and cost problems^18^. Additionally, the priority in the early stages of cardiac arrest is to treat the clinical problem that caused it and provide hemodynamic treatment, making it difficult to perform these tests. Moreover, different patient illness categories and clinical departments, along with the limited availability of multidisciplinary treatment and intensive care teams in some hospitals, hinder the implementation of standard integrated treatment after cardiac arrest^11^. In cases of patients with end-stage diseases or irreversible clinical deterioration, intensive treatment after cardiac arrest may not offer meaningful benefits due to limited life expectancy. Similarly, for terminally ill patients or those in the process of dying, decisions regarding life-sustaining treatment should prioritize the best interest of the patient. These decisions should be made based on fundamental medical ethics principles, including informed consent, respect for autonomy, non-maleficence, beneficence, and justice^19^. To ensure informed decision-making regarding the initiation, continuation, or withdrawal of life-sustaining treatment (WLST) for unconscious patients recovering from cardiac arrest, the patient's guardian and attending physician play crucial roles. Early prognosis assessment is vital, and caregivers should receive comprehensive explanations regarding the patient's prognosis and treatment plan. The CASPRI and GO-FAR scores are useful tools focused on pre-cardiac arrest and cardiac arrest factors, aiding in early assessment and explanation of various clinical indicators related to patient survival and neurological prognosis. However, it is essential not to base decisions solely on these indicators, especially considering discontinuing life-sustaining treatment in the early stages of cardiac arrest. Instead, they can serve as reference points to determine whether resuscitation is appropriate when spontaneous circulation has been achieved but full recovery has not been attained despite treatment, and the patient is in a terminal state. For a more comprehensive assessment of the patient's overall prognosis, these scores should be used in conjunction with other diagnostic tools to determine neurological prognosis. This approach will facilitate the judgment of the patient's overall condition and guide appropriate treatment decisions.

This study has some limitations. First, there is a possibility that the information obtained by retrospectively analyzing patient medical records may be different from the actual situation at the time of cardiac arrest. Secondly, our study was conducted at a single center with a relatively small participant pool. Unlike certain countries that have registries for IHCA patients, Korea lacks a comprehensive multi-center research registry. Variances in guidelines for life-sustaining treatment decisions and emergency response protocols exist between hospitals and countries. The overall incidence of IHCA reflects patient illness burden, a facility's ability to detect deterioration, and the effectiveness of its resuscitation response system, all of which can vary among individual hospitals^14^. For these reasons, the outcomes and survival rates of IHCA may be biased. Thus, future hospital-based multicenter studies and research based on policy investigations are needed to review and confirm the results of the present study. Third, while the AUROC is a helpful tool for assessing predictive ability, it might not fully address differences at specific decision points or consider factors like misclassification costs and population variations. It's important to complement AUC with other metrics and clinical insights for a well-rounded evaluation. Fourth, variability in DNR and WLST decisions among hospitals and their impact on IHCA incidence rates raise concerns. In 2018, in South Korea, a legal process for the creation of WLST documents was introduced. Prior to this, there was no formal procedure for decisions regarding CPR or discontinuation of life-sustaining treatment. As a result, surrogated DNR orders were also included^20^. While DNR orders are often prepared in advance for high-risk patients, their exclusion could limit insights into various critical illnesses and clinical conditions' effects on neurological prognosis. Considering factors like poor prognosis, legal concerns, and resource utilization, medical staff should carefully consider DNR documentation. Fifth, Targeted temperature management (TTM) was not included in this study due to its limited use for in-hospital cardiac arrest patients at this study’s hospital, except in the emergency medicine department. The decision was influenced by factors like clinical complexities, cost concerns, and uncertain outcomes, especially for critically ill patients. TTM's significant impact on patients' neurological prognosis makes its absence in this study potentially lead to different findings than research that includes TTM as a treatment approach^21^. Finally, the data used in this study were collected before the start of the coronavirus pandemic in Korea. However, there was only one case of morbidity owing to the coronavirus disease.

## Conclusion

IHCA often occurs in patients in an already clinically deteriorated state. Thus, clinical treatment related to comorbidities and the prognosis of the disease, in addition to treatment and neurological prognosis after cardiac arrest, are important in establishing a treatment plan. The CASPRI scores can be used as auxiliary tools that aid decision-making in the initial stages of cardiac arrest treatment.

## Methods

### Study design and study population

This was a retrospective study conducted at a tertiary general hospital from September 2016 to August 2021. Adult patients who had IHCA and achieved ROSC for at least consecutive 20 min were enrolled in this study. Patients younger than 18 years old, those who had a cardiac arrest during surgery or procedures, those who had another cardiac arrest within 24 h, those who had written a letter of intent to discontinue life-sustaining treatment, and those who had a cardiac arrest while in the emergency room were excluded from this study. Data were extracted from the quarterly IHCA and CPR reports of the hospital. Records related to the occurrence of cardiac arrest were primarily reviewed by the CPR committee. Two authors independently verified these reports using electronic medical records. Using some modifications of the revised *2020 American Heart Association guidelines* and an *Utstein-style template*, patient information, and in-hospital factors, such as age, sex, time and place of cardiac arrest, illness category, actual CPR execution time, initial rhythm, presence of defibrillation, and time required to defibrillate; and CPR-related factors, such as recovery of spontaneous circulation and extracorporeal resuscitation, were investigated. CPC score at discharge was set as the primary outcome in this study. While the CPR committee focuses on resuscitation and ROSC data reported quarterly in the CPR registry, it is mandatory to include discharge outcomes, including survival and neurological prognosis. Two authors meticulously reviewed medical records and confirmed the final CPC scores based on this information. CPC score of 1 or 2 is classified as a good neurological condition, whereas a score of 3, 4 or 5(death or brain death) is considered a poor neurological condition^22–24^. The secondary outcomes were survival-to-discharge and normal neurological status or minimal neurological damage at discharge (CPC score of 1). The CASPRI score is based on a patient’s age, initial rhythm, CPR time, cranial neurological status 24 h prior to cardiac arrest, location of cardiac arrest, and the presence of mechanical ventilation, renal dysfunction, liver dysfunction, sepsis, malignancy, and hypotension^6^. The GO-FAR system is a summed score that consists of 13 pre-arrest variables^7^. For the GO-FAR score, clear consciousness at the time of admission is judged as the most important neurological predictor (-15 points). Severe trauma, stroke, malignancy, sepsis, and hospitalization for non-cardiac medical diseases result in poor prognosis. In addition, liver dysfunction, transfer from a nursing hospital, hypotension, renal dysfunction or dialysis, respiratory failure, presence of pneumonia, and age are considered predictors. As shown in the table, several items are duplicated^7^. In cases where a poor prognosis was anticipated, it was common for patients to receive WLST early. Following the American Heart Association (AHA) guidelines, cases where WLST was administered within 72 h of neurological prognosis assessment were termed "early WLST" and were included in regression analysis^21^.

### Ethical declarations

This study was conducted in accordance with the Declaration of Helsinki and received approval from the Institutional Review Board (IRB) of Pusan National University Yangsan Hospital (IRB No. 05-2022-172). Given the retrospective and observational nature of this minimal-risk study, the requirement for obtaining informed consent from all subjects was waived by the IRB in accordance with applicable national regulations. This decision is in line with the guidelines set forth in the “Ethical Guidelines for Medical and Health Research Involving Human Subjects” published by the Ministry of Health and Welfare, Republic of Korea^25^. The analysis was performed using anonymized clinical data to ensure subject privacy and confidentiality.

### Ethical approval declarations

This study was approved by the institutional review board (IRB) of Pusan National University Yangsan Hospital and waived under IRB approval because of the retrospective and observational nature of this study. Patient’s information was anonymized and removed prior to analyses. (IRB no. 05-2022-172).

### Statement of human and animal rights

This article does not contain any studies with animals performed by any of the authors.

## Statistical analysis

The collected data were analyzed using SPSS Statistics version 27.0 (IBM Corp., Armonk, NY, USA) and R software version 4.2.0. The components of the CASPRI and GO-FAR scores were summarized using descriptive statistics. The chi-square and Fisher’s exact tests were used to compare the categorical variables. Categorical data are expressed as numbers and percentages. The Shapiro–Wilk and Kolmogorov–Smirnov tests were used to test the normality of the distributions of continuous variables. Normally distributed variables were analyzed using student’s t-test or analysis of variance; otherwise, the Mann–Whitney U-test was used. Continuous data are expressed as mean ± standard deviation or median (interquartile range [IQR]). Receiver operating characteristic curves and areas under the curves were used to evaluate the efficacy of the CASPRI and GO-FAR scores for the prediction of neurological prognosis and survival. In addition to the CASPRI and GO-FAR scores, we analyzed the MEWS^26^ and the qSOFA score^27^, which are used to predict transfer to the intensive care unit during hospitalization and cardiac arrest and in-hospital mortality, respectively. Also, we used the Youden J statistic in the R, pROC package to determine the cut-off value. Furthermore, we utilized DeLong's test in R to assess whether there is a statistically significant difference between each ROC curve of the models. To confirm the degree of correlation in the prediction of neurological prognosis, univariate analysis of all independent variables, including the factors related to the scores, the basic characteristics of the patients according to the Utstein-style template, underlying diseases, and pre-cardiac arrest/cardiac arrest-related factors except for CASPRI, GO-FAR, MEWS, and qSOFA scores, was performed. Thereafter, we performed multi-variable regression analysis using variables with the dependent variable on bi-variable analysis (*p* < 0.1), and pre-defined variables that made clinical sense. The final variables in the model were determined by backward elimination (likelihood ratio) selection. The odds ratio and 95% confidence intervals of the results were compared through multivariable logistic regression analysis of the components of the CASPRI and GO-FAR scores (Supplementary Tables).

### Supplementary Information

Supplementary Tables.

Supplementary Table S1.

## Data Availability

The datasets generated during and/or analysed during the current study are available from the corresponding author on reasonable request.
