# Supplementary material for: Predicting in-hospital cardiac arrest outcomes: CASPRI and GO-FAR scores
Source: Sci Rep. 2023 Oct 23;13:18087. doi: 10.1038/s41598-023-44312-2 (PMC10593798; doi:10.1038/s41598-023-44312-2)
Supplement: Supplementary file 1 — Supplementary Tables. [file 41598_2023_44312_MOESM1_ESM.docx]

Table S1 The Cardiac Arrest Survival Postresuscitation In-hospital (CASPRI) Score

| 1. Age group, y | Points |  | 4. Hospital location | Points |
| --- | --- | --- | --- | --- |
| <50 | 0 |  | Telemetry unit | 0 |
| 50-59 | 0 |  | Intensive care | 1 |
| 60-69 | 1 |  | Nonmonitored unit | 3 |
| 70-79 | 2 |  | 5. Duration of resuscitation, min | Points |
| ≥80 | 4 |  | <2 | 0 |
| 2. Initial arrest rhythm; | Points |  | 2-4  5-9 | 0  3 |
| VF/VT time to defibrillation |  |  |  |  |
| ≤2 minutes | 0 |  | 10-14 | 5 |
| 3 minutes | 0 |  | 15-19 | 6 |
| 4-5 minuts | 2 |  | 20-24 | 6 |
| >5 minuts | 3 |  | 25-29 | 6 |
| Pulseless electrical activity | 6 |  | ≥30 | 8 |
| Asystole | 7 |  | Factors present prior to arrest | points |
| 3. Prearrest CPC score | Points |  | 6. Mechanical ventilation | 3 |
| 1 | 0 |  | 7. Renal insufficiency | 2 |
| 2 | 2 |  | 8. Hepatic insufficiency | 4 |
| 3 | 9 |  | 9. Sepsis | 3 |
| ≥4 | 9 |  | 10. Malignant disease | 4 |
|  |  |  | 11. Hypotension | 3 |

Reprinted from Chan et al [6] where a detailed description of the score's interpretation is presented. CPC indicates cerebral performance score; VF/VT, ventricular fibrillation or ventricular tachycardia.

Table S2. Good Outcome Following Attempted Resuscitation (GO-FAR) Score to Predict Neurologically Intact Survival After In-Hospital Cardiopulmonary Resuscitation

| Variable | Score |
| --- | --- |
| Neurologically intact at admission | -15 |
| Major trauma | 10 |
| Acute stroke | 8 |
| Metastatic or hematologic cancer | 7 |
| Septicemia | 7 |
| Medical non-cardiac diagnosis | 7 |
| Hepatic insufficiency | 6 |
| Admission from skilled nursing facility | 6 |
| Hypotension or hypoperfusion | 5 |
| Renal insufficiency including dialysis | 4 |
| Respiratory insufficiency | 4 |
| Pneumonia | 1 |
| Age (years) |  |
| 70-74 | 2 |
| 75-79 | 5 |
| 80-84 | 6 |
|  | 11 |

Reprinted from Ebell MH et al [7], where a detailed description of the score's interpretation is presented.

Table S3. Definitions of predictor variables, adapted to the American heart association’s Get With the Guidelines-Resuscitation registry.

| Variables | Definition |
| --- | --- |
| Acute, Non-Stroke Neurologic Disorder | evidence of decreased mental status, delirium, or coma not due to acute stroke within 4 hours of the cardiac arrest |
| Hepatic Insufficiency | Evidence within 24 hours of cardiac arrest of either of the following: 1. direct bilirubin > 2mg/dL and AST > 2 times upper limit of normal, 2. liver cirrhosis. |
| Renal insufficiency | Serum creatinine >2 mg/dL or Requiring ongoing dialysis or extracorporeal filtration therapies within 24 hours of cardiac arrest. |
| Respiratory Insufficiency | Evidence of acute or chronic respiratory insufficiency within 4 hours of cardiac arrest, defined by any of the following: 1. PaO2 / FiO2 ratio 40 per minute or < 5 per minute 5. Non-invasive ventilation (e.g., mask or nasal CPAP or BiPAP) 6. Invasive ventilation (e.g., T-piece, external respirator) |
| Hypotension or hypoperfusion | Any evidence of hypotension within 4h of the event, defined as any of the following: SBP <90 or MAP < 60mmHg; vasopressor or inotropic requirement after volume expansion (except for dopamine <3ug/kg/min); intraaortic balloon pump |
| Septicemia | Documented bloodstream infection in which antibiotic therapy has not yet been started or is till ongoing |
| Metastatic or hematologic cancer | Any solid tissue malignancy with evidence of metastaaasis or any blood-borne malignancy |
| Pneumonia | Documented diagnosis of active pneumonia, in which antibiotic therapy has not yet been started or is still ongoing |
| Major trauma | Evidence of multi-system injury or single-system injury associated with shock or altered mental status during the current hospitalization |
| Acute stroke | Documented diagnosis of an intracranial or intraventricular hemorrhage or thrombosis during the current admission |
| Metabolic derangement | Arterial pH < 7.3 or > 7.5; Lactate >2.5 mmol/L; Blood glucose |
| Electrolyte abnormality | Sodium < 125 or > 150 mEq/L; Potassium < 2.5 or > 6 mEq/L |

Reprinted from Ebell MH et al [7], where a detailed description of the score's interpretation is presented
