# Supplementary material for: Predicting in-hospital cardiac arrest outcomes: CASPRI and GO-FAR scores
Source: Sci Rep. 2023 Oct 23;13:18087. doi: 10.1038/s41598-023-44312-2 (PMC10593798; doi:10.1038/s41598-023-44312-2)
Supplement: Supplementary file 2 — Supplementary Table S1. [file 41598_2023_44312_MOESM2_ESM.pdf]

Online Supplementary Table S1

Table S1. Main inclusion/exclusion criteria

Main inclusion criteria:

Patients eligible for inclusion in the study had to fulfill all of the following criteria:

1. Patients must give written informed consent before any study-related assessment is performed.
2. Patients must be ≥ 18 years of age.
3. Patients must have a diagnosis of Rheumatoid Arthritis, based on the ACR 1987 criteria.
4. Patients must have active RA at baseline (Visit 2):
    - ≥ 6 swollen joints (of 66 joints assessed)
    - ≥ 6 tender joints (of 68 joints assessed)
    - CRP ≥ 10 mg/L
      OR ESR ≥ 28 mm/1st hour
5. Patients must be seropositive for rheumatoid factor (RF) and/or have antibodies to cyclic citrullinated peptide (anti-CCP) at Visit 1.
6. Patients must have inadequate response or intolerance to non-biologic DMARDs, and one or up to three TNF antagonists:
7. Patients must be on a stable dose of MTX (7.5-25 mg per week). They must have received MTX for at least 4 months with 25 mg/week as the maximal dose, and with a stable dose for 4 weeks prior to randomization.
8. Patients must have received folic acid or equivalent for at least 4 weeks prior to randomization at a stable dose ≥ 5 mg per week.
9. Patients must have stopped etanercept, certolizumab pegol and adalimumab for at least 4 weeks, infliximab and golimumab for at least 8 weeks before randomization.
10. If taking glucocorticoids, patients must be on stable dose of maximally 10 mg/d prednisone or equivalent over the past 2 weeks before randomization.

Main exclusion criteria

Patients eligible for inclusion in the study must not had fulfilled any of the following criteria:

1. RA patients with functional status class IV classified according to the ACR 1991 revised criteria.
2. Patients taking high potency opioid analgesics (e.g. methadone, hydromorphone, morphine).
3. Female patients nursing (lactating / breast-feeding), pregnant or planning of pregnancy within 12 months after the last infusion of study drug, where pregnancy is defined as the state of a female after conception and until the termination of gestation, confirmed by a positive hCG laboratory test (cut-off as defined by the central laboratory).

4. Women of child-bearing potential, defined as all women physiologically capable of becoming pregnant, UNLESS they are using a highly effective method of birth control (i.e. one that results in a less than 1% per year failure rate when used consistently and correctly, such as implants, injectables, combined oral contraceptives, and intrauterine devices (IUDs)). Periodic abstinence (e.g. calendar, ovulation, symptothermal, post-ovulation methods) is not acceptable.
5. Patients who have had any therapy with intra-articular injections (e. g. glucocorticoid) required by a flare up to 4 weeks before randomization.
6. Patients with underlying metabolic, hematologic, renal, hepatic, pulmonary, neurologic, endocrine, infectious or gastrointestinal conditions, which in the opinion of the investigator immunocompromizes the patient and/or places the patient at unacceptable risk by receiving immunomodulatory therapy within the study – especially patients with clinical history of Felty's Syndrome.
7. Patients with significant medical problems, including but not limited to the following: uncontrolled hypertension (≥ 160/95 mmHg), congestive heart failure (New York Heart Association status of class III or IV).
8. Patients with total WBC count < 3000/µL, platelets < 100,000/µL, neutrophils < 1,500/µL or hemoglobin < 8.5 g/dL (85 g/L) at Visit 1 and/or Visit 2.
9. History of clinically significant liver disease or liver injury as indicated by abnormal liver function tests such as AST, ALT, or alkaline phosphatase. Any single parameter may not exceed 3 x upper limit of normal (ULN). A single parameter elevated up to and including 3 x ULN should be re-checked once more as soon as possible, and in all cases, at least prior to randomization.
10. Patients with history of renal trauma, glomerulonephritis, a single kidney or a calculated Glomerular Filtration Rate (calculated as per 4-variable MDRD-formula) < 60 ml/min/1.73 m^2.
11. History of lymphoproliferative disease or any known malignancy or history of malignancy within the past 5 years prior to randomization (except non-melanoma skin cancer which has been treated with no evidence of recurrence in the past 3 months, carcinoma in situ of the cervix, polyps (removed) in the colon with non-invasive malignancy).
12. Patients with any medical, neurologic or psychiatric condition, which in the investigator's opinion would preclude the patient from completing all protocol requirements.
13. Patients with other inflammatory diseases which might confound the evaluation of the efficacy (e.g. Crohn's disease, ulcerative colitis).
14. Patients with ongoing, chronic infectious disease or history of recurrent infectious diseases and with a history of active tuberculosis within the previous 2 years should not be included. Patients with an evidence of tuberculosis infection as defined by either a positive PPD skin test (the size of induration will be measured after 48-72 hours, and a positive result is defined as an induration of ≥ 5 mm or according to local guidelines) or a positive QuantiFERON TB-Gold test may enter the study if further work-up (according to local practice/guidelines) establishes conclusively that the patient has no evidence of active tuberculosis. The appropriate test will be chosen according to local

guidelines/regulations, if applicable. If presence of latent tuberculosis is established then treatment according to local guidelines must have been initiated.

15. Active systemic infections during the two weeks (exception: common cold) prior to randomization.
16. Recent arthroplasty (within 3 months prior to randomization).
17. Patients with a current severe progressive or uncontrolled disease which in the judgment of the clinical investigator renders the patient unsuitable for the study.
18. History of allergy (medication history) to any of the compounds used in the study.
19. Hypersensitivity to any of the study medication excipients or to murine proteins.
